# Alterations of gut microbiota‐derived metabolites in gestational diabetes mellitus and clinical significance

**DOI:** 10.1002/jcla.24333

**Published:** 2022-03-13

**Authors:** Yajie Gao, Haimin Chen, Jialin Li, Shuaijun Ren, Zhenglun Yang, Yuping Zhou, Rongrong Xuan

**Affiliations:** ^1^ 47862 Department of Obstetrics and Gynecology The Affiliated Hospital of Medical School of Ningbo University Ningbo China; ^2^ 47862 Key Laboratory of Applied Marine Biotechnology of Ministry of Education Ningbo University Ningbo China; ^3^ 47862 School of Medicine Ningbo University Ningbo China; ^4^ 47862 Department of Gastroenterology The Affiliated Hospital of Medical School of Ningbo University Ningbo China; ^5^ Institute of Digestive Disease of Ningbo University Ningbo China

**Keywords:** bile acids, gestational diabetes mellitus, gut microbiota‐derived metabolites, short‐chain fatty acids, targeted metabolomics, trimethylamine N‐oxide

## Abstract

**Background:**

The change in the characteristics of the gut microbiota is linked to gestational diabetes mellitus (GDM). However, whether and how the gut microbiota‐derived metabolites change in GDM is uncertain. Here, we aimed to determine associations between the gut microbiota‐derived metabolites and GDM.

**Methods:**

Using targeted metabolomics approaches, 7 types of short‐chain fatty acids (SCFAs), 38 types of bile acids (BAs), and 5 types of trimethylamine N‐oxide (TMAO), and its derivatives of serum samples were obtained from pregnant women with GDM (*n* = 24), and healthy pregnant controls (HC, *n* = 28) were detected to identify the metabolic signature of GDM to investigate the potential biomarkers. Moreover, we assessed the associations between gut microbiota‐derived metabolites and clinical parameters.

**Results:**

In our study, the gut microbiota‐derived metabolites signatures were significantly different between GDM and HC. Quantitative results showed the levels of isobutyric acid, isovaleric acid, valeric acid, caproic acid, GUDCA, THDCA + TUDCA, and LCA‐3S were significantly higher in GDM, but the level of TMAO and its derivatives did not change significantly. Some altered gut microbiota‐derived metabolites were significantly correlated with glucose and lipid levels. Receiver‐operating characteristic (ROC) analysis of generalized linear models showed that gut microbiota‐derived metabolites may be potential biomarkers of GDM.

**Conclusion:**

This study highlights gut microbiota‐derived metabolites alterations in GDM and correlation of the clinical indicators, which provides a new direction for future studies aiming to novel serum biomarker for early detection or target of drug therapy of GDM.

## Graphical abstract

Using targeted metabolomics approaches, identify the gut microbiota‐derived metabolites signature of GDM to investigate the potential biomarkers and assess the clinical significance.

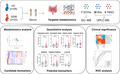

## 1 INTRODUCTION

Gestational diabetes mellitus (GDM) is one of the most common pregnancy complications, which defined as glucose intolerance resulting in hyperglycemia, with onset or first recognition during pregnancy. According to reports, the global incidence of GDM is 2.3%–17.5%.^1^ With the development of society and economy, changes in lifestyles and increasing emphasis on GDM screening, the detection rate of GDM is increasing year by year. Previous studies have indicated that GDM women were at higher risk of adverse perinatal outcomes, including hypertension, preeclampsia, infection, preterm delivery, macrosomia, increased cesarean rates, premature rupture of membranes (PROM), perinatal mortality, and neonatal metabolic complications.^2^ These adverse outcomes in GDM have led clinicians to implement various strategies including fetal monitoring and induction of labor, which is a major threat to maternal and fetal health. Several risk factors for GDM have already been identified, including maternal age, family history of diabetes, prepregnancy obesity, and multiple pregnancies.^3^ In recent years, the correlation of gut microbiota with GDM has become a research hotspot. Several macrogenomic studies showed that the diversity of gut microbiota in patients with GDM is lower than that in healthy pregnant women. *Ruminococcaceae*, *Parabacteroides distasonis*, *Prevotella*, *Desulfovibrio*, *Megamonas*, and *Phascolarctobacterium* are enriched in the gut microbiota of pregnant women with GDM. These microbiotas are related to the metabolic pathways of glucose and lipid metabolism and insulin signal transduction. Gut microbiotas can use the nutrients of the host to produce microbial metabolites, finally forming host–microbe metabolic axis between host and gut microbes. This axis plays an important role in nutrition metabolism and immune homeostasis and ultimately affects the overall metabolism of the host. Gut microbiota‐derived metabolites act as information messengers between the gut microbiotas and host cells, including short‐chain fatty acids (SCFAs), bile acids (BAs), choline, tryptophan, indole derivatives, and trimethylamine N‐oxide (TMAO), etc.^4^ Studies on rodents have shown potential mechanisms of interaction with the gut microbiome, including regulating glucose metabolism, increasing short‐chain fatty acids, enhancing the permeability of lipopolysaccharides and the interaction with bile acids.^5^ In addition, human studies have proved the evidence for these hypotheses.^6^ However, the interaction between the imbalance of the gut microbiota of GDM patients and host metabolism is still unclear. Therefore, studying the changes in gut microbiota‐derived metabolites of GDM will help to further understand the mechanism of gut microbiota involved in the occurrence and development of GDM and has important guiding value for the early prediction, diagnosis, and timely treatment of GDM.

Metabolomics detection can capture the metabolic changes associated with the disease, identify metabolic markers in the development of the disease, and help discover new etiology and pathogenesis. Hou et al.^7^ offered significant biochemical parameters and perinatal data changes in free fatty acids, bile acids, branched‐chain amino acids, organic acids, lipids, and organooxygen compounds in 131 GDM cases compared with 138 controls by fasting serum metabolite analysis. In a pilot UPLC‐MS study, Liu et al.^8^ explored and identified 35 metabolites in serum metabolites between women with GDM and healthy controls during and after pregnancy, which involved in important metabolic pathways such as glycine, serine, threonine, steroid hormone biosynthesis, tyrosine metabolism, glycerophospholipid metabolism, and fatty acid metabolism that contribute to GDM progression. In addition to blood (plasma or serum), urine, amniotic fluid, placenta, and newborn's meconium also provide a rich metabolomic profile for GDM studies. The identification of signature metabolites can be used for disease diagnosis, therapeutic response assessment, or even predicting susceptibility to the disease. However, highly sensitive and specific metabolic biomarkers for detecting GDM in early pregnancy by targeting metabolomic probes of serum gut microbiota‐derived metabolites levels are unavailable.

The aim of this study was to perform targeted gut microbiota‐derived metabolites (SCFAs, BAs, TMAO, and its derivatives) analysis of serum samples from pregnant women with GDM and healthy pregnant controls, to identify serum biomarkers and their metabolic pathways, and correlation with clinical indicators, laying the foundation for early diagnosis, early warning, and even reversal of pregnancy outcomes in GDM.

## 2 MATERIALS AND METHODS

### 2.1 Study population and sample collection

This was a cross‐sectional study in 52 pregnant women (24 pregnant women with GDM and 28 healthy pregnant women) in the third trimester of pregnancy who gave birth in the Affiliated Hospital of Medical School of Ningbo University between November 2020 and February 2021. The inclusion criteria were as follows: (1) single birth, (2) no history of hypertension, diabetes, cardiovascular and cerebrovascular diseases, and metabolic diseases before pregnancy, and (3) no other complications of pregnancy. The exclusion criteria were someone who took antibiotics, probiotics, and prebiotics within 1 month prior to sampling, or who had diarrhea and other gastrointestinal symptoms. GDM was diagnosed using an Oral Glucose Tolerance Test (OGTT) performed between 24 and 28 gestational weeks using the International Association of Diabetes and Pregnancy Study Group (IADPSG) criteria [FBG ≥ 5.1 mmol/L (92 mg/dl), 1‐h post‐OGTT ≥ 10.0 mmol/L (180 mg/dl) or 2‐h post‐OGTT ≥ 8.5 mmol/L (153 mg/dl)].^9^ The pregnant women whose glucose levels were normal in OGTT were designated as healthy controls (HC). All participants had signed a written informed consent, which had been approved by the Institutional Review Board (IRB) of the Affiliated Hospital of Medical School of Ningbo University with the code KY20201124.

We collected the clinical information of the two groups including age, vital signs, height, weight, prepregnancy BMI, gravidity, parity, and pregnancy outcomes. Results of biochemistry tests including triglycerides (TG), total cholesterol (TC), low‐density lipoprotein (LDL) cholesterol, high‐density lipoprotein (HDL) cholesterol, white blood cell count (WBC), hemoglobin (Hb), neutrophil percentage (NEU%), C‐reactive protein (CRP), alanine aminotransferase (ALT), aspartate aminotransferase (AST), albumin, total bile acid (TBA), blood urea nitrogen (BUN), creatinine (Cr), BUN/Cr, and uric acid (UA) were also collected. Participants’ blood samples were collected after 8–10 h of fasting. Sample transfer centrifugation (1000 *g* for 10 min at 4°C) and separation of serum were completed within 1 hour. Final serum samples were stored at −80°C until retrieval for targeted metabolomics analysis.

### 2.2 Biochemical analysis

The complete blood count analysis was performed using a hematology system (Mindray BC 6800), which was based on a combination of light scatter, electrical impedance, fluorescence, light absorption, and electrical conductivity methods to produce complete blood cell analyses. TG, TC, LDL, HDL, ALT, AST, albumin, TBA, BUN, Cr, BUN/Cr, and UA concentrations were determined by the enzymatic colorimetric method (Beckman Coulter AU5800).

### 2.3 Quality control

Equal volume of samples from each experimental sample was mixed as a quality control (QC) sample. The blank samples were the blank matrix of the experimental samples with the same pretreatment process as for the experimental samples.

### 2.4 Targeted GC‐MS analysis of SCFAs

Targeted analysis of 7 types of short‐chain fatty acids (acetic acid, propanoic acid, butyric acid, isobutyric acid, valeric acid, isovaleric acid, and caproic acid) in serum samples using the gas chromatography‐mass spectrometry (GC‐MS) method. Briefly, 20 μl of serum sample mixed with 15% phosphoric acid (50 μl), 75 μg/ml of internal standard solution (isocaproic acid, 10 μl), and ether (140 μl) were precisely pipetted for pretreatment, derivatization, and extraction of target analytes. Samples were centrifuged at 13000*g* at 4°C for 10 min and 150 µl of the upper organic layer was collected for analysis. Samples were analyzed by Thermo TRACE 1310‐ISQ LT GC‐MS (Thermo). The sample was injected in split mode(10:1), and helium (1 ml/min) was used as carrier gas. SCFAs were performed using an HP‐INNOWAX column (30 m × 0.25 mm, 0.25 μm; Agilent) with an electrospray ionization (ESI) source in positive ionization mode, and small molecules were measured by gas chromatography‐mass spectrometer (GC‐MS). The temperatures of chromatographic inlet, ion source, transfer line, and quadrupole mass spectrometer were maintained at 250, 230, 250, and 150°C. The starting temperature of the programmed temperature rise is 90°C, then increased to 120°C at 10°C/min and then to 150°C at 5°C/min. Finally, the temperature is increased to 250°C at 25°C/min for 2 min. The obtained extracts were assayed for analytes by GC‐MS.

### 2.5 Targeted UPLC‐MS analysis of BAs

Targeted analysis of 38 kinds of BAs (alloLCA, LCA, isoLCA, NorDCA, 6‐ketoLCA, 12‐ketoLCA, 7‐ketoLCA, beta‐UDCA, DCA, CDCA, UDCA, HDCA, NorCA, DHCA, 7,12‐diketoLCA, 6,7‐diketoLCA, alpha‐MCA, UCA, beta‐MCA, CA, ACA, beta‐CA, GLCA, GHDCA, GCDCA, GUDCA, GDCA, LCA‐3S, GCA, TLCA, THDCA + TUDCA, TDCA, TCDCA, TCA, T‐alpha‐MCA, THCA, T‐beta‐MCA, and CDCA‐G) in serum samples using the ultra‐performance liquid chromatography‐mass spectrometry (UPLC‐MS) method. Measure a proper amount of sample into a 2‐ml EP tube, accurately add methanol (300 μl, −20°C) to precipitate the protein, vortex for 60 s, centrifuge at 12,000 rpm at 4°C for 10 min, take the supernatant, and concentrate it with a vacuum concentrator at room temperature until completely dry. Pipette accurately reconstitute the sample with methanol (100 μl, −20°C), vortex and shake for 30 s, take 90 μl of the supernatant, and add it to the detection bottle. The sample was injected in 5 μl. BAs were performed using an ACQUITY UPLC BEH C18 column (2.1 × 100 mm, 1.7 μm; Waters) with an ESI source in negative ionization mode. The ion source temperature was 500°C, the ion source voltage was −4500 V, the collision gas is 6 psi, the curtain gas was 30 psi, and the atomization gas and auxiliary gas were both 50 psi. Multiple reaction monitoring (MRM) was used for scanning. Column temperature was at 40℃, eluent A was 0.01% formic acid water, and eluent B was acetonitrile. The solvent gradient was set as follows: 0–4 min, 25% B; 4–9 min, 25%–30% B; 9–14 min, 30%–36% B; 14–18 min, 36%–38% B; 18–24 min, 38%–50% B; 24–32 min, 50%–75% B; 32–35 min, 75%–100% B; and 35–38 min, 100%–25% B. The flow rate was 0.25 ml/min.

### 2.6 Targeted UPLC‐MS analysis of TMAO and its derivatives

Targeted analysis of TMAO and its derivatives (choline, betaine, TMAO, creatinine, and L‐carnitine) in serum samples using the UPLC‐MS method. 20 μl of serum sample mixed with add 10 μl of internal standard solution, and then add 750 μl of 1% formic acid‐acetonitrile solution, vortex for 30 s, centrifuge at 12,000 rpm for 5 min at 4°C, take 500 μl of supernatant, filter through 0.22 μm of membrane, and filter add the liquid to the test bottle. Samples were performed using an ACQUITY UPLC BEH HILIC column (2.1 × 100 m, 1.7 μm; Waters) with an ESI source in positive ionization mode, injection volume 5 μl, column temperature 40℃, mobile phase A‐acetonitrile, B‐water (containing 0.1% formic acid), and 10 mM of ammonium formate at a flow rate of 0.4 ml/min. The solvent gradient was set as follows: 0–1 min, 80% A; 1–2 min, 80%–70% A; 2–2.5 min, 70% A; 2.5–3 min, 70%–50% A; 3–3.5 min, 50% A; 3.5–4 min, 50%–80% A; and 4–6 min, 80% A. The ion source temperature was 500℃, the ion source voltage was 5000 V, the collision gas was 6 psi, the curtain gas was 30 psi, and the atomization gas and auxiliary gas were both 50 psi. Multiple reaction monitoring (MRM) was used for scanning.

### 2.7 Metabolomics data analysis

Univariate and multivariate analysis methods, which is partial least‐square discriminant analysis (PLS‐DA) and agglomerate hierarchical clustering were conducted to examine the potential differential metabolites and was plotted by the Pheatmap package in R language (version 3.3.2). A univariate analysis (*t* test) was applied to calculate the statistical significance, and *p*‐value < 0.05 were considered differential metabolites. Statistically significant values of correlation between differential metabolites were calculated by cor.mtest in R language. Additionally, the receiver–operator characteristics (ROC) analysis was performed, and area under the curve (AUC) was used to evaluate metabolites diagnostic capabilities. Spearman's correlation was used to assess the significance correlations between metabolites and clinical parameters. Correlation coefficients (r) and *p*‐value were calculated. The metabolic pathways were studied using the Kyoto Encyclopedia of Genes and Genomes (KEGG; Kanehisa Laboratories) database.

### 2.8 Statistical analysis

SPSS 26.0 (SPSS Inc.) was used for statistical analysis. The Student's *t* test or Mann–Whitney *U* test were performed to evaluate the difference among groups for continuous variables, and non‐normally distributed data were tested by the Wilcoxon's rank‐sum test. The continuous variables were presented as mean ± standard error (SD) unless otherwise. The chi‐squared test or Fisher's exact test was conducted for categorical variables to compare the difference. A two‐sided *p*‐value < 0.05 was considered statistically significant.

## 3 RESULTS

### 3.1 Characteristics of the study population

A total of 52 participants (24 GDM and 28 HC) were enrolled. The maternal and fetal general characteristics of subjects were listed in Table 1, and the clinical characteristics were summarized in Table 2. Compared with the HC, the GDM group showed glucose and lipid metabolism disturbance. Fasting OGTT glucose level, and 1‐ and 2‐h post‐OGTT glucose levels at 24–28 weeks’ gestation were significantly elevated in GDM (all *p*‐value < 0.05). Meanwhile, the levels of TG, TC, and LDL in the GDM group were remarkably higher than those in HC, which were opposite to the level of HDL (all *p*‐value < 0.05). There were no statistical group differences for other variables, such as prepregnancy BMI, parity, fetal birth weight, blood pressure, and inflammation indicators (all *p*‐value < 0.05).

**TABLE 1:** General characteristics of study population

Characteristic	GDM (*n* = 24)	HC (*n* = 28)	*p*‐Value
Baseline characteristics of the subjects			
Age (years)	30.54 ± 4.67	28.61 ± 2.81	0.096^b^
Gestational weeks (weeks)	39.27 ± 0.75	39.22 ± 0.79	0.265^b^
Height (cm)	161.17 ± 6.54	163.25 ± 5.14	0.204^a^
Weight (kg)	57.79 ± 9.44	59.17 ± 8.64	0.762^b^
Prepregnancy BMI (kg/m^2^)	22.23 ± 3.38	22.16 ± 2.62	0.985^b^
Maternal weight gain (kg)	13.67 ± 4.26	14.20 ± 5.01	0.682^a^
Gravidity	1.92 ± 1.25	1.96 ± 1.17	0.715^b^
Parity, *n* (%)			
Primiparous (1 birth)	16 (0.67)	15 (0.54)	0.403^c^
Multiparous (≥2 births)	8 (0.33)	13 (0.46)	
Pregnancy outcomes			
Delivery week (weeks)	39.42 ± 0.80	39.36 ± 0.86	0.298^a^
Fetal birth weight (kg)	3.43 ± 0.38	3.50 ± 0.44	0.512^a^
Mode of delivery, *n* (%)			
Vaginal	17 (0.71)	21 (0.75)	0.764^c^
Caesarean section	7 (0.29)	7 (0.25)	

^a^ Derived from the Student's *t* test.

^b^ Derived from the Mann–Whitney *U* test.

^c^ Derived from the chi‐squared test or Fisher's exact test.

**TABLE 2:** Clinical characteristics of study population

Characteristic	GDM (*n* = 24)	HC (*n* = 28)	*p*‐Value
Systolic blood pressure (mm Hg)	119.38 ± 11.49	120.25 ± 9.43	0.521^a^
Diastolic blood pressure (mm Hg)	73.46 ± 6.81	74.86 ± 7.27	0.480^a^
Fasting glucose, OGTT (mmol/L)	4.72 ± 0.58	4.32 ± 0.32	**0.005** ^a^
1 h glucose, OGTT (mmol/L)	9.29 ± 1.61	7.71 ± 1.72	**0.002** ^a^
2 h glucose, OGTT (mmol/L)	8.60 ± 1.09	6.62 ± 1.10	**<0.001** ^a^
TG (mmol/L)	3.88 ± 1.23	3.09 ± 0.98	**0.026** ^a^
TC (mmol/L)	7.01 ± 1.08	6.20 ± 1.40	**0.045** ^a^
LDL (mmol/L)	3.80 ± 0.81	3.22 ± 0.82	**0.025** ^a^
HDL (mmol/L)	1.89 ± 0.36	2.21 ± 0.47	**0.021** ^a^
WBC (×10^9^/L)	9.49 ± 2.27	10.15 ± 2.47	0.313^b^
Hb (g/L)	128.42 ± 9.96	126.96 ± 12.49	0.649^a^
NEU (%)	75.27 ± 6.01	78.05 ± 6.92	0.131^a^
CRP (mg/L)	7.62 ± 10.84	5.51 ± 5.77	0.797^b^
ALT (U/L)	10.22 ± 4.26	9.39 ± 3.24	0.567^b^
AST (U/L)	18.91 ± 4.43	19.75 ± 3.44	0.148^b^
Albumin (g/L)	37.29 ± 7.86	37.9 ± 6.49	0.208^b^
TBA (μmol/L)	3.33 ± 1.65	2.71 ± 1.37	0.145^b^
BUN (mmol/L)	3.74 ± 1.11	3.30 ± 0.74	0.098^a^
Cr (μmol/L)	47.49 ± 8.63	46.49 ± 6.20	0.663^a^
BUN/Cr	0.08 ± 0.16	0.07 ± 0.16	0.139^a^
UA (μmol/L)	325.09 ± 71.18	327.18 ± 77.49	0.921^a^

Abbreviations: ALT, alanine aminotransferase; AST, aspartate aminotransferase; BUN, blood urea nitrogen; Cr, creatinine; CRP, C‐reactive protein; GDM, gestational diabetes mellitus; Hb, hemoglobin; HDL, high‐density lipoprotein; LDL, low‐density lipoprotein; NEU%, neutrophil percentage; OGTT, oral glucose tolerance test; TBA, total bile acid; TC, total cholesterol; TG, triacylglycerides; UA, uric acid; WBC, white blood cell count. Bold‐faced values means *p*<0.05.

^a^ Derived from the Student's *t* test.

^b^ Derived from the Mann–Whitney *U* test.

### 3.2 The gut microbiota‐derived metabolites signature in GDM were altered

**FIGURE 1:**
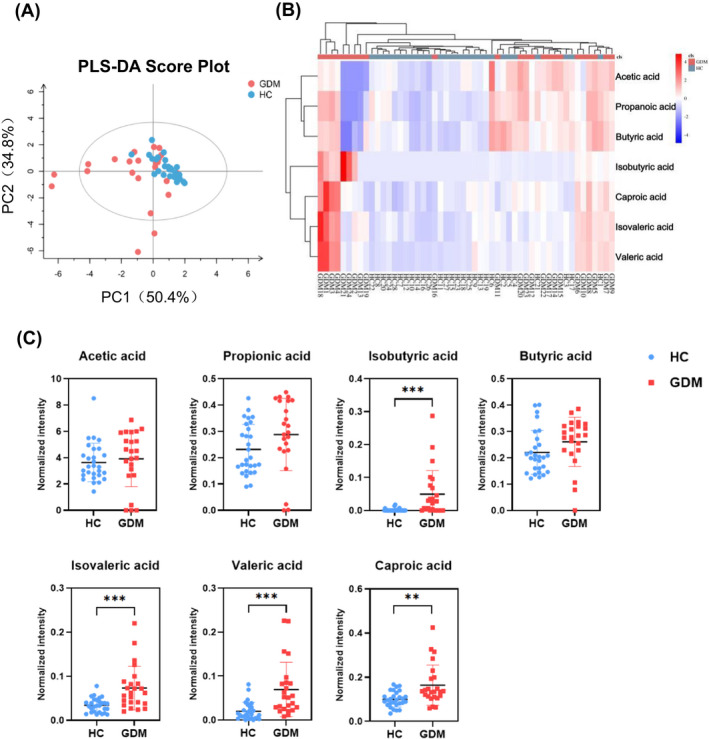
Changes in serum short‐chain fatty acids (SCFAs) of GDM and HC. (A) The PLS‐DA scores plot of SCFAs in serum samples of the GDM group (red) and HC group (blue). The horizontal axis represents the predicted score of the first component, which explained 50.4% of the between‐group variations. The vertical axis represents the orthogonal principal component score, which explained 34.8 of the within‐group variations. R2X = 0.853, R2Y = 0.301, Q2 = 0.245. (B) Heatmap analysis of SCFAs between the GDM and HC groups. Hierarchical clustering analysis was performed on each group, different color regions represent different clustering group information. Red indicates relatively high kurtosis values, and blue indicates relatively low kurtosis values. (C) Quantitative comparison of serum SCFAs between the GDM and HC groups. **p < 0.01, ***p < 0.001

Recent years, accumulating evidences supported that SCFAs might affect glucose metabolism. In this study, GC‐MS platform was used for metabolite measurement, 7 types of SCFAs were quantified. PLS‐DA was used as a supervised method to get an overview of the data and to detect trends of metabolome alteration between the two groups. Based on the PLS‐DA scores scatterplot (Figure 1A), we observed a clear separation between GDM and HC. In order to define the relationship between the samples more intuitively and comprehensively, and to evaluate the differences in the expression patterns of metabolites in different samples, we use hierarchical clustering analysis on each group of samples to accurately screen marker metabolites and explore changes in related metabolic processes. Therefore, we generated a metabolite heatmap, which revealed considerable differences between GDM and HC (Figure 1B). Quantitative analysis results showed 4 types of SCFAs were significantly elevated in the GDM group, including isobutyric acid, isovaleric acid, valeric acid, and caproic acid (Figure 1C). Among them, isobutyric acid showed the most significant change, which was 19.363 times higher than HC. However, there were no statistical differences on levels of acetic acid, propanoic acid, and butyric acid in serum between the GDM and HC groups.

**FIGURE 2:**
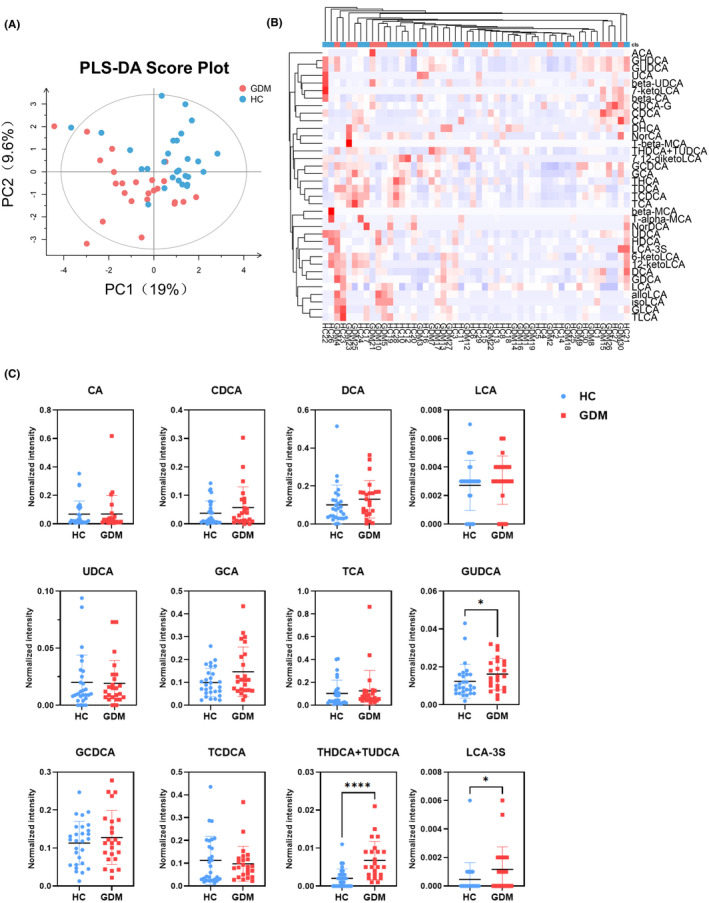
Changes in serum bile acids (BAs) of GDM and HC. (A) The PLS‐DA scores plot of BAs in serum samples of the GDM group (red) and HC group (blue). The horizontal axis represents the predicted score of the first component, which explained 19% of the between‐group variations. The vertical axis represents the orthogonal principal component score, which explained 9.6% of the within‐group variations. R2X = 0.294, R2Y = 0.289, Q2 = −0.17. (B) Heatmap analysis of BAs between the GDM and HC groups. (C) Quantitative comparison of serum BAs between the GDM and HC groups. *p < 0.05, ****p < 0.0001

Bile acids are now considered to be signaling molecules that coordinate glucose and energy metabolism. We used UPLC‐MS platforms to analysis of 38 types of BAs in serum samples. PLS‐DA scores scatterplot showed a discriminative trend of BAs between the GDM and HC groups (Figure 2A). However, heatmap showed no obvious difference in the BAs level (Figure 2B). Quantitative analysis results showed GUDCA, THDCA + TUDCA (taurohyodeoxycholic acid + tauroursodeoxycholic acid), and LCA‐3S were significantly increased in the GDM group (Figure 2C). However, there was no statistical difference of other BAs between the GDM and HC groups (Figure S1).

**FIGURE 3:**
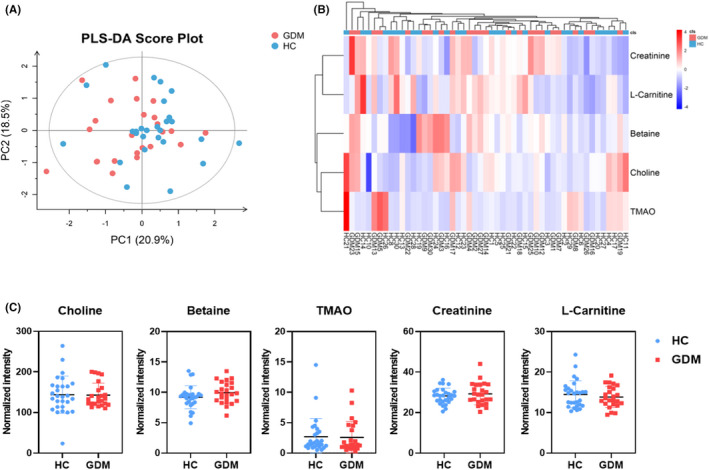
Changes in serum trimethylamine N‐oxide (TMAO) and its derivatives of GDM and HC. (A) The PLS‐DA scores plot of TMAO and its derivatives in serum samples of the GDM group (red) and HC group (blue). The horizontal axis represents the predicted score of the first component, which explained 20.9% of the between‐group variations. The vertical axis represents the orthogonal principal component score, which explained 18.5% of the within‐group variations. R2X = 0.442, R2Y = 0.0898, Q2 = −0.36. (B) Heatmap analysis of TMAO and its derivatives between the GDM and HC groups. (C) Quantitative comparison of serum TMAO and its derivatives between the GDM and HC groups

Although there was a large amount of evidence supporting the involvement of TMAO in glucose metabolism, there were few existing literatures on TMAO and GDM. In this study, there were no significant differences in serum TMAO and its derivatives’ concentrations between the GDM and HC groups (Figure 3A–C).

### 3.3 Maternal glucose values and blood lipids level was correlated with altered gut microbiota‐derived metabolites

**FIGURE 4:**
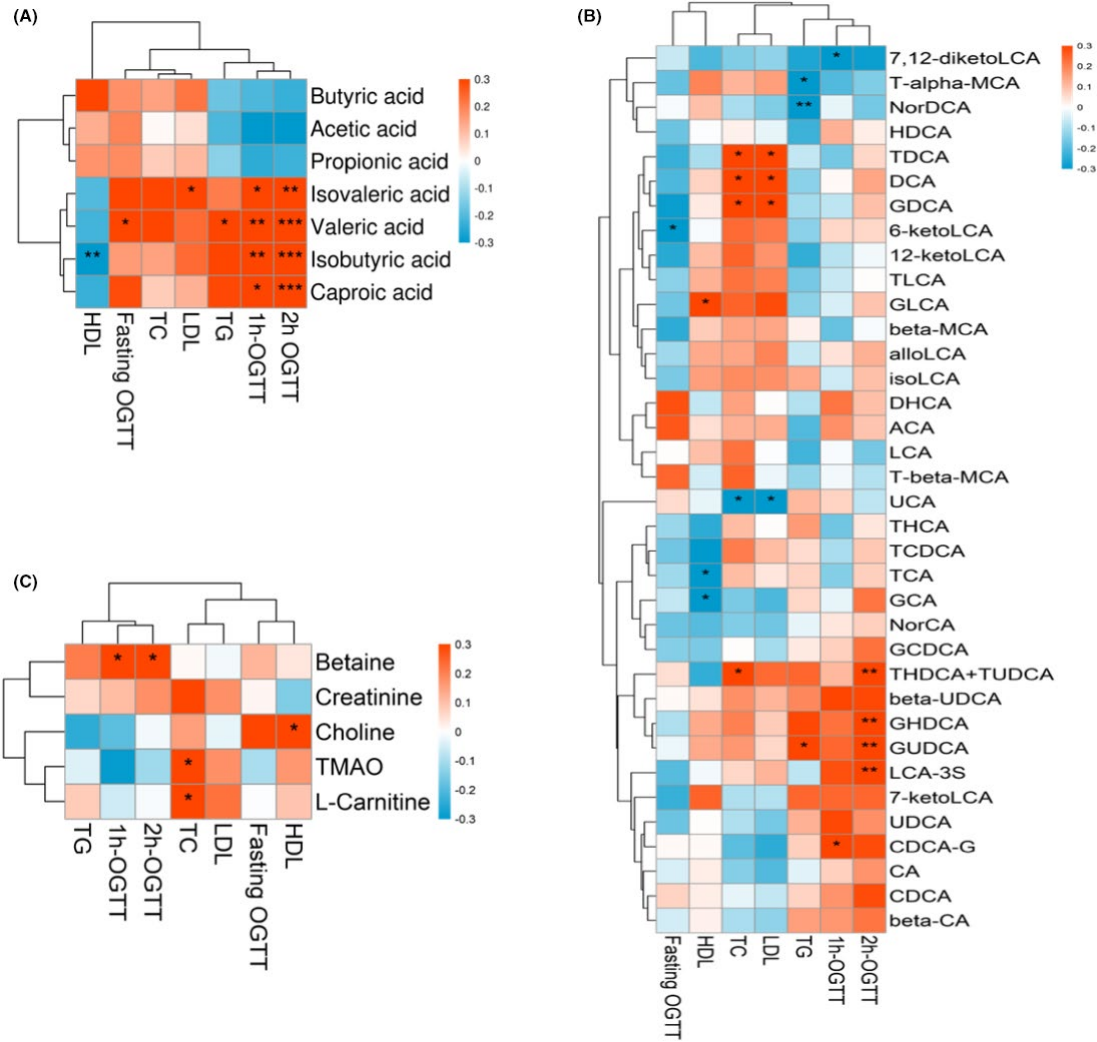
Associations among glucose values, blood lipids level, and altered gut microbiota‐derived metabolites. *p < 0.05, **p < 0.01, ***p < 0.001

To further explore potential correlations of key clinical indexes with altered gut microbiota‐derived metabolites in GDM, random forest analysis was performed in Figure 4. At 24–28 weeks’ gestation, 1‐ and 2‐h post‐OGTT blood glucose values all showed substantial positive correlations with isobutyric acid, isovaleric acid, valeric acid, caproic acid, THDCA + TUDCA, GUDCA, and LCA‐3S. Among them, valeric acid was also positively correlated with fasting OGTT blood glucose values. These results were consistent with the results of quantitative analysis. In addition, betaine, CDCA‐G, and GHDCA were also positive correlations with 1‐ and 2‐h post‐OGTT blood glucose values, although their concentrations were not observed and changed in this study, while 7,12‐diketoLCA and 6‐ketoLCA were negatively correlated with 2‐h post‐OGTT blood glucose values and fasting OGTT, respectively. Significantly, we found that gut microbiota‐derived metabolites were correlated with blood lipid levels. Such as, valeric acid and GUDCA were positively correlated with TG level, contrary to T‐alpha‐MCA and NorDCA. Isovaleric acid, TDCA, DCA, and GDCA were positively correlated with the LDL level. Isobutyric acid, TCA, and GCA were negatively correlated with HDL level, contrary to GLCA. These findings suggest changes in blood glucose and blood lipids in patients with GDM are related to altered gut microbiota‐derived metabolites.

**FIGURE 5:**
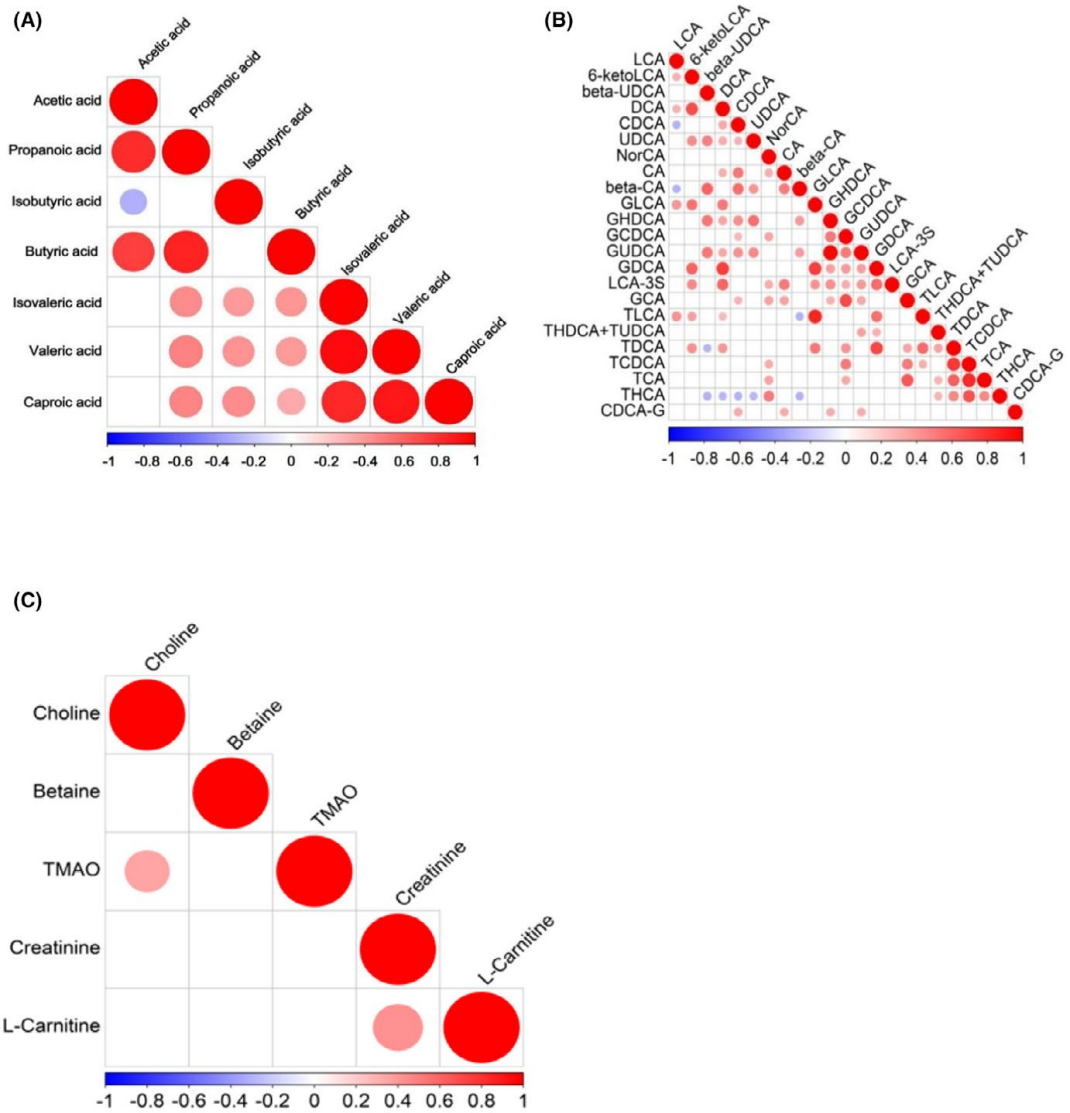
Associated heatmap of gut microbiota‐derived metabolites. (A–C) When the linear relationship of the two metabolites is enhanced, the correlation coefficient tends to 1 or −1. The correlation is a maximum of 1, a complete positive correlation (red), a correlation of −1, and a complete negative correlation (blue)

The metabolite correlation heatmap showed that there were also correlations between each metabolites (Figure 5A–C). Through correlation analysis, we observed that isobutyric acid was significantly negatively correlated with acetic acid. Propanoic acid, butyric acid, and isobutyric acid were significantly positive correlated with caproic acid, isovaleric acid, and valeric acid. Besides, circulating THDCA + TUDCA was positively associated with beta‐UDCA, GHDCA, and GUDCA. GUDCA was positively associated with beta‐UDCA, DCA, CDCA, UDCA, beta‐CA, GHDCA, and GCDCA. LCA‐3S was positively associated with 6‐ketoLCA, DCA, NorCA, CA, GLDCA, GHDCA, GCDCA, GUDCA, and GDCA. The possible reason is that the gut microbiota‐derived metabolites can be transformed into each other under certain conditions. For example, primary bile acids (CA, CDCA) are transformed into secondary bile acids under the action of intestinal bacteria. Although there had no changes in TMAO and its derivatives were observed in this study, the metabolites correlation heatmap showed there may be some correlation between TMAO and its derivatives, such as TMAO is positively correlated with choline, and carnitine is positively correlated with L‐carnitine.

### 3.4 Gut microbiota‐derived metabolites related to lipid metabolism, amino acid metabolism, and glucose metabolism

**FIGURE 6:**
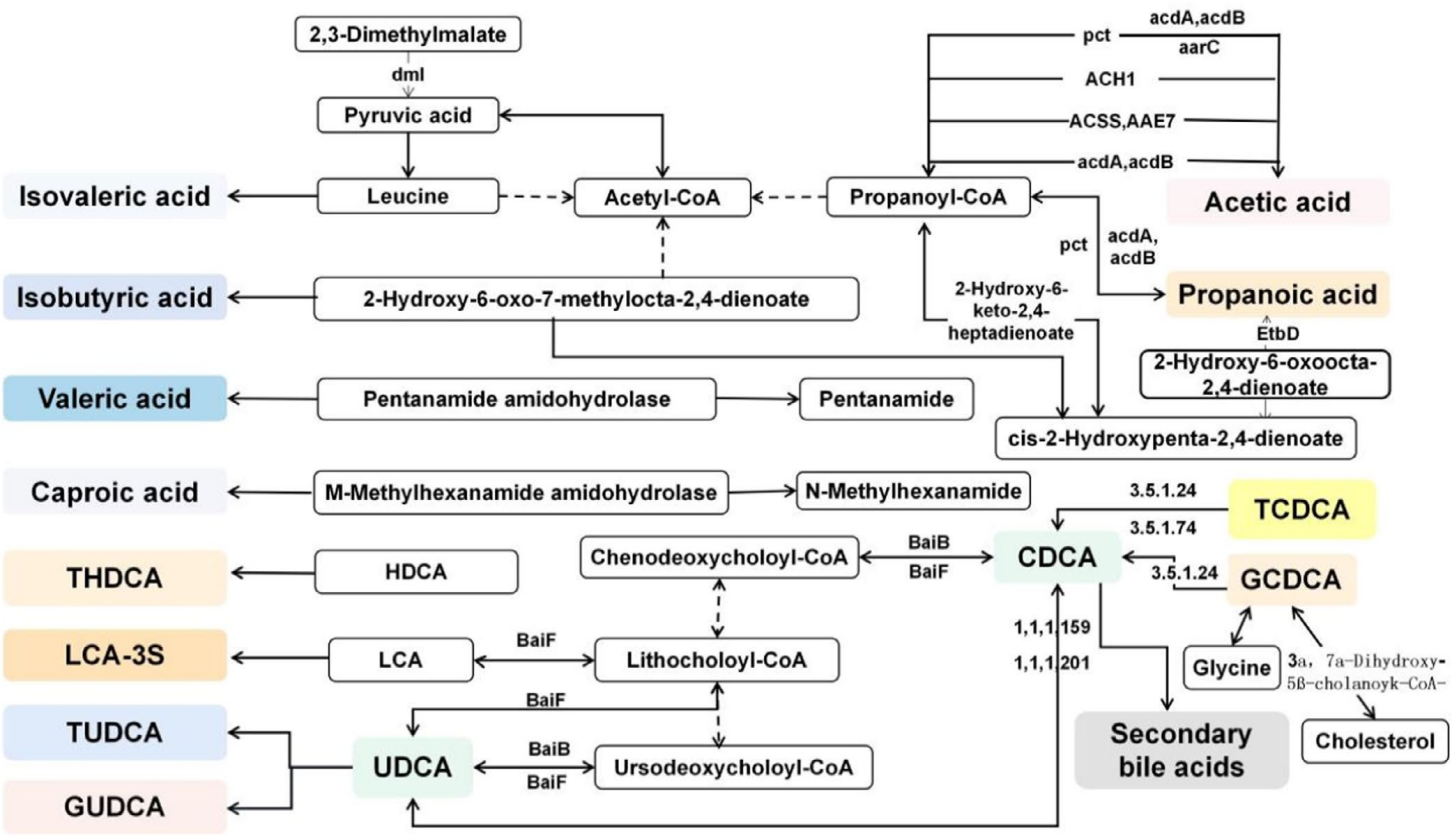
Differential metabolite KEGG pathway enrichment map

KEGG pathway enrichment prediction of differential metabolites was then performed, and a map of the gut microbiota‐derived metabolites metabolic pathways was constructed (Figure 6). The biosynthesis of acetyl‐CoA, leucine metabolism, biosynthesis of pentanamide, and biosynthesis of N‐methylhexanamide were all found to be statistically different between GDM and HC. In addition, bile acids can modulate cholesterol synthesis. These modifications to the host metabolism represented the influence of the gut microbiota‐derived metabolites on lipid metabolism, amino acid metabolism, and glucose metabolism.^10^

### 3.5 Gut microbiota‐derived metabolites markers can discriminate GDM

**FIGURE 7:**
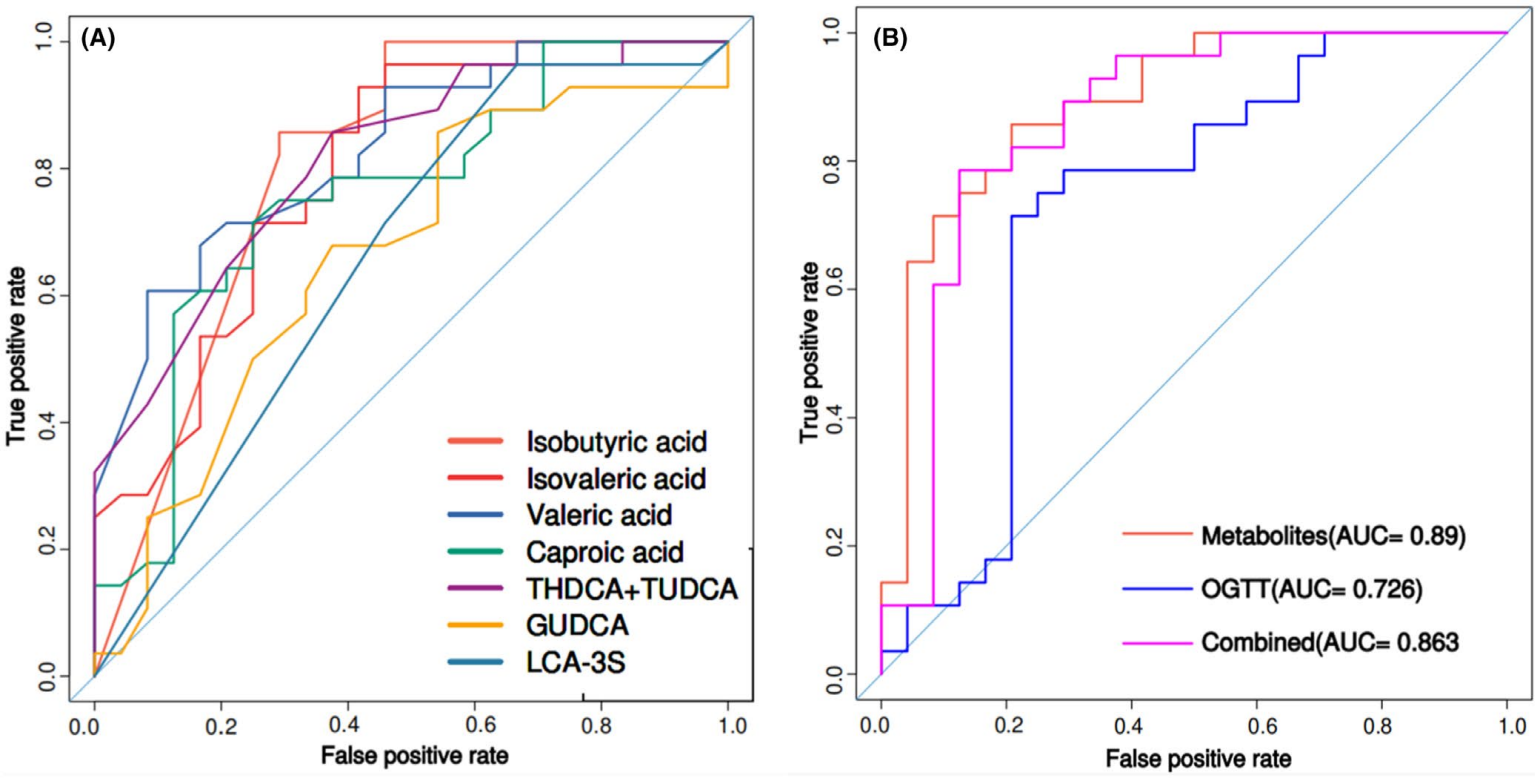
Area under the curves of differential metabolites between the GDM and HC groups. (A) ROC curves were prepared for different metabolites. The ordinate is the sensitivity, which represents the true positive rate, and the abscissa is the specificity, which represents the false positive rate. (B) Random forest analysis was used to quantify diagnostic performance of biomarker panels. Individual marker panels could distinguish patients with the GDM and HC groups with an area under the curve (AUC) ranging from 0.726 to 0.890. AUC, area under the curve; ROC, receiver‐operating characteristic

Random forest analysis was used to explore whether gut microbiota‐derived metabolites markers can be used to discriminate subjects with GDM from HC. Based on the differential metabolites (isobutyric acid, isovaleric acid, valeric acid, caproic acid, GUDCA, THDCA + TUDCA, and LCA‐3S), binary logistic regression analysis was performed on the GDM and HC groups. In the ROC graph, the AUC indicates the diagnostic potentials of the metabolites as unique biomarkers for identification of GDM and control, as shown in Figure 7A. Summary of ROC curve parameters was shown in Table 3. ROC analysis of generalized linear model showed that isobutyric acid, isovaleric acid, valeric acid, caproic acid, GUDCA, THDCA + TUDCA, and LCA‐3S may be potential biomarkers of GDM. We could accurately distinguish GDM patients from healthy controls, as indicated by the area under the receiver‐operating curve (AUC), which had a value up from 0.661 to 0.831. Among the strongest discriminatory features, valeric acid (VA) had the greatest impact (AUC = 0.831, 95%CI: 0.723–0.939). These results highlight the early diagnosis potential of these gut microbiota‐derived metabolites markers. Moreover, we created a combinatorial marker panel composed of these 7 biomarkers, which AUC ranging from 0.726 to 0.890 (Figure 7B), and these results highlight the early diagnosis potential of the new combination markers.

**TABLE 3:** ROC analysis parameters of differential metabolites between the GDM and HC groups

	Sensitivity (%)	Specificity (%)	AUC	95% CI
Valeric acid	71.4	79.2	0.831	0.723–0.939
THDCA + TUDCA	78.6	66.7	0.814	0.700–0.928
Isobutyric acid	85.7	70.8	0.806	0.693–0.919
Isovaleric acid	71.4	75.0	0.797	0.673–0.920
Caproic acid	71.4	75.0	0.754	0.618–0.891
GUDCA	67.9	62.5	0.663	0.510–0.816
LCA‐3S	71.4	54.2	0.661	0.525–0.797

## 4 DISCUSSION

In patients with GDM, the secretion of pancreatic islets is restricted or the function of islet is impaired, so the balance of glucose metabolism cannot be maintained, resulting in increased blood glucose levels. At present, GDM can use the OGTT method for early screening and diagnosis, which is currently recognized as the "gold standard," but this method is time‐consuming and can cause discomfort for pregnant women. Moreover, the current screening and diagnostic criteria for GDM are not uniform around the world, which may lead to insufficient diagnosis and poor management of the disease. Therefore, searching for early GDM diagnosis and effective intervention means are very important. Thanks to the rapid development of metabolomic research technology, the metabolite research may become a breakthrough point of GDM diagnosis and effective intervention. Several studies have reported that GDM exist metabolites’ alteration disorders of glucose metabolism, lipid metabolism, and amino acid metabolism.^11^ The intestine affects the absorption and utilization of glucose and also affects the metabolism and absorption of fat, which is closely related to the occurrence of GDM. Therefore, in this study, we identified several different gut microbiota‐derived metabolites in the serum, including short‐chain fatty acids, bile acids, and TMAO, which are closely related to the disease process of GDM.

Short‐chain fatty acids (SCFAs) are organic fatty acids with 1–6 carbon atoms, which are mainly produced by fermentation of indigestible carbohydrates (dietary fiber) and intestinal flora through different metabolic pathways. SCFAs can be present in a small amount in the circulation and provide energy to peripheral tissues as substrates for hepatic glyconeogenesis or adipocyte adipogenesis. Among SCFAs, acetic acid, propionic acid, and butyric acid have the highest content, accounting for more than 85%. In organisms, SCFAs have a variety of biological functions such as providing energy, maintaining water and electrolyte balance, improving intestinal blood circulation, immune regulation, gene expression regulation, and affecting the metabolic function of the offspring.^12^ The total concentration of SCFAs depends on the diet, type, and number of the host microbiotas, and the time spent in the gastrointestinal tract. Generally, *Firmicutes*, especially *Faeculus*, *Rossella*, and *Bifidobacterium* produce butyrate, while *Bacteroides* produces acetate and propionate. Depending on the diet, the total concentrations of SCFAs decreased from 70 to 140 mM in the proximal colon to 20 to 70 mM in the distal colon, which is associated with the increased availability of carbohydrate and water in the proximal colon.^13^ In the colon and rectum, 95% of the produced SCFAs are rapidly absorbed by the colon cells, while the remaining 5% are secreted in the feces. Thus, fecal SCFAs excretion provides little information about the actual gut SCFA metabolism. Therefore, we detected the levels of SCFAs in the serum of pregnant. SCFAs appear to have a beneficial influence glucose metabolism by normalizing blood glucose levels and increasing glucose treatment. SCFAs regulate host lipid and glucose metabolism through the G protein‐associated receptors (GPR41 and GPR43).^14^ In addition, SCFAs also activates enteroendocrine L cells and stimulates glucagon‐like peptide‐1 (GLP‐1) and intestinal hormone peptide YY (PYY). GLP‐1 promotes insulin secretion and inhibits the release of glucagon from the pancreas. In animal models, elevation at body PYY levels was observed after SCFA injection within the colon.^15^ Therefore, altered levels of SCFAs can lead to disturbed host metabolism, leading to obesity and diabetes. In recent years, multiple clinical cohort studies have shown that SCFAs levels are closely related to type 2 diabetes (T2DM). GDM, as a metabolic disease during pregnancy, often combined with maternal overweight or obesity, leading to changes in gut microbial composition, diversity, and SCFAs ratio. A high‐fat, low‐fiber diet in GDM women may alter normal gut microbiota composition, leading to elevated Firmicutes and Faecalibacterium, resulting in excessive production of SCFAs. In addition, SCFAs may increase the glycolysis/glyconeogenesis pathway and inhibit insulin signaling in peripheral tissues, leading to hyperglycemia in GDM.

Our research results showed valeric acid, caproic acid, isobutyric acid, and isovaleric acid were significantly higher in the GDM group. However, there were no statistical differences on levels of acetic acid, propanoic acid, and butyric acid in serum between the GDM and HC groups. Valeric acid is a short‐chain fatty acid with 5 carbon atoms. It is produced by the microbial metabolism of aminoproline and hydroxyproline and lactic acid and propionic acid.^16^ Intestinal flora is the most likely source of valeric acid. Previous studies have determined that valeric acid is a ligand for G protein‐coupled receptors (GPCRs), which has certain effects on metabolism, immunity, and blood pressure regulation.^17^ However, studies have also shown that the increase in valeric acid concentration is correlated with the levels of inflammation markers (C‐reactive protein and white blood cell count).^18^ Inflammatory response mediates insulin resistance, which plays an important role in the occurrence and development of GDM. Therefore, we speculate that the increase in serum valeric acid levels in GDM patients may be related to the inflammatory state of the gut microbiota. Caproic acid is directly generated by some bacteria that form the microbiota, especially *Prevotella*. Studies have shown that these bacteria increased in GDM patients, leading to increased caproic acid production.^19^ In addition, we also observed some branched‐chain amino acids that can be used to distinguish GDM from the control group using targeted metabolomics. Isobutyric acid and isovaleric acid are branched‐chain fatty acids (BCFAs), which are mainly produced during the fermentation of branched‐chain amino acids (valine, leucine, and isoleucine) by the intestinal flora mainly. *Bacteroides* and *Clostridium* are mainly responsible for the fermentation process of BCFAs.^20^ In vitro intestinal models, high‐protein and low‐complex carbohydrate diets (such as Western diets) can lead to higher BCFAs levels, which has been further confirmed in certain dietary interventions.^21^ In vitro experiments on rat and human adipocytes have shown that BCFAs inhibit cAMP‐mediated lipolysis and insulin‐stimulated lipogenesis, and isobutyric acid can also enhance insulin‐stimulated glucose uptake.^22^ In this study, the levels of BCFAs in the serum of GDM patients increased. The possible reasons are that the reduced utilization of fermentable carbohydrates in the intestinal flora may have facilitated the shift to more protein fermentation, leading to increase in BCFA production.

Bile acids (BAs) are small molecules synthesized by cholesterol in liver cells, which are now thought to be signaling molecules that coordinate blood glucose, lipid, and energy metabolism. The changes of BAs are closely related to metabolic abnormalities, such as T2DM, insulin resistance, and obesity. BAs alter metabolism by activating certain receptors, including the farnesian class X receptor (FXR), pregnant class X receptors, and G protein‐coupled receptors (GPCRs). Large cohorts from China evaluated the association between total BAs levels and GDM in early pregnancy, where elevated total BAs content may be associated with an increased risk of GDM.^23^ The association between BAs and GDM is rare, and careful analysis of the effects of specific BAs on GDM is more rare. In this study, GUDCA, THDCA + TUDCA, and LCA‐3S were significantly increased in the GDM group. TUDCA has been shown to reduce the ER stress associated with elevated blood glucose levels in diabetic patients by inhibiting caspase activation, upregulation of UPR, and inhibition of reactive oxygen species, exhibits antidiabetic activity.^24^ These results are contrary to our findings. Glycoursodeoxycholic acid (GUDCA) is a glycine‐conjugated form of UDCA. A recent study reported that GUDCA can act as an intestinal farnesol X receptor antagonist, significantly reducing weight gain and restoring glucose intolerance and insulin resistance in a diet‐induced obesity mouse model.^25^ In addition, increased serum GUDCA levels are associated with decreased hemoglobin A1c and waist circumference in patients with T2DM.^26^ The above evidence indicates that GUDCA has potential metabolic beneficial effects. However, in our study, GUDCA level in the GDM group was increased and positively correlated with blood glucose and triglyceride levels. LCA is the most toxic bile acid because of its strong hydrophobicity. Under normal circumstances, the amount of LCA in the body is very tiny. Sulfation of BAs reduces the cholestasis of DCA, CA, and CDCA. After sulfation of LCA, hydrophilicity increases and toxicity decreases. Sulfated BAs have a low level of serum, but the content of sulfated BAs in urine is 40%–70% of the total urine bile acids and is not affected by diet. Therefore, the conclusions of this study need to be validated using a larger group of participants and other types of samples.

Trimethylamine N‐oxide (TMAO) is one of the most important gut microbiota‐derived metabolites. Gut microbiota can use choline‐rich substances to produce trimethylamine (TMA), and then, TMA activated by hepaflavin monooxygenase generates TMAO. The amount of TMAO production is related to the type of human gut microbiota. This fully illustrates the high relevance of TMAO to the development of GDM. In recent years, several clinical cohort studies have shown that the body TMAO levels are closely related to T2DM, and animal studies have found that TMAO can aggravate impaired glucose tolerance and hyperglycemia by blocking liver insulin signaling and promoting an adipose tissue inflammatory response.^27^ Consistently, reducing plasma TMAO could improve glucose and lipid homeostasis in mice by using FMO3 inhibition.^28^ Further studies found that mice with FMO3 deletion were protected from obesity caused by a high‐fat diet.^29^ However, to date, the role of choline and its metabolites in the pathogenesis of GDM had not been extensively studied. An exploratory NMR metabolomic study of 54 samples explored the relationship between TMAO and GDM, suggesting lower plasma TMAO concentrations in GDM subjects.^30^ However, an observational study of 866 pregnant women (433 cases of GSM and 4331:1 matched controls by age, gestational week, and birth) showed a significantly positive association between plasma TMAO concentrations and the risk of GDM.^31^ A recent review suggests that circulating TMAO levels are affected by multiple factors, including gut microbiota composition and activity, liver function and excretion, gut blood‐barrier function, and diet.^32^ However, there was no significant changes in the TMAO level of GDM in our study. This may be attributed to differences in the composition and function of the gut microbiota. Some bacterial families from *Firmicutes* and *Proteobacteria* are powerful choline and carnitine consumers capable of TMA synthesis by expressing specialized enzymes. In addition, the expression and activity of other biochemical factors such as liver FMO3 can also affect TMAO levels. Thus, highly variable plasma levels in both disease and nondisease states are caused by differences in intestinal bacterial composition, and it is not necessarily a marker in the disease process. Therefore, further assessment of TMAO status is needed in future studies, which should include collecting duplicate samples and determining averages at different times, and needs to be verified by more different populations, while thinking about exactly how it correlates with GDM.

Moreover, we carefully examined the relationship between differential metabolites and the clinical information of the samples, including blood glucose and lipid levels. Maternal blood glucose values and blood lipid levels were found to be correlated with altered gut microbiota‐derived metabolites in the GDM. Lipid metabolism is essential for healthy pregnancy and development. Dyslipidemia is a common phenomenon during pregnancy, and it is considered to be a physiological mechanism to provide fuel and nutrition for the fetus. During normal pregnancy, plasma lipid mass spectra including TG, TC, HDL, and LDL levels change significantly due to insulin resistance, lipoprotein synthesis, and increased lipolysis in adipose tissue, which is called dyslipidemia during pregnancy (DLP). The regulation of dietary type, gut microbiota, and lipid metabolism may be linked by affecting the levels of metabolic hormones. A high‐fat diet in GDM women disrupts the gut microbiota, resulting in the growth of butyrate‐producing bacteria (mainly *Firmicutes* and the *Bacterium faecalis*), and increases SCFA production. SCFAs overactivate glycolysis and glyconeogenesis pathways and suppress the insulin response in peripheral tissues, leading to diabetes. In our study, the random forest analysis results of blood lipid levels and SCFAs suggested a positive correlation between valeric acid and TG levels, isovaleric acid and LDL levels, and isobutyric acid and HDL levels. These findings suggest that SCFAs are highly correlated with maternal blood lipid levels. Several studies point to an important role of SCFAs in cholesterol levels. Acetate, the most abundant SCFAs in peripheral circulation, is a substrate for cholesterol and promotes cholesterol synthesis. The reduced ratio of acetate to propionate may lead to a decrease in serum lipids.^33^, ^34^ However, their mechanism of action has conflicts with results. This study showed no significant difference in the levels of acetate and propionate between the GDM and HC groups; further studies suggest that conclusive evidence was critical for entry into human studies.

Meanwhile, bile acids are closely related to lipid metabolism. In this study, we observed GUDCA, TDCA, DCA, and GDCA were positively correlated with serum lipid level, contrary to T‐alpha‐MCA and NorDCA. Moreover, TCA and GCA were negatively correlated with HDL level, contrary to GLCA. There is a key receptor in BAs metabolism, farnesol X receptor (FXR), which is the liver‐intestinal bridge of BAs metabolism and can regulate the synthesis and reabsorption of BAs. FXR is distributed in a variety of tissues, especially as the liver and gut are the most distributed and more active. Zhang et al.^35^ found that Gly‐MCA acts as an antagonist to inhibit intestinal FXR, thereby tearing host liver lipid metabolism and improving obesity‐associated metabolic disorders. Oral cannabinoids inhibits intestinal bacterial BSH activity, resulting in the accumulation of bound bile acids, inhibiting intestinal FXR‐FGF15 signaling, and thus ultimately reducing cholesterol level.^36^ An 8‐week parallel randomized controlled trial demonstrated that Mediterranean dietary intervention in overweight and obese subjects reduced plasma cholesterol and altered the gut microbiota and fecal bile acids, significantly reducing fecal total bile acids, including primary and secondary bile acids. In particular, DCA and LCA in the stool significantly decreased at 4 and 8 weeks after the intervention.^37^ In addition, common buckwheat protects against high‐fat diet‐induced nonalcoholic fatty liver disease (NAFLD) associated with dyslipidemia in mice. The addition of common buckwheat significantly regulated the biosynthesis of primary BAs and altered the structure of the gut microbiota, thus improving lipid metabolism.

To evaluate the diagnostic ability of gut microbiota‐derived metabolites, we used random forest analysis to find that separate metabolic markers could effectively discriminate the GDM patient from HC. The result revealed gut microbiota‐derived metabolites may be used as potential serum biomarkers for predicting GDM. Among them, valeric acid has the largest AUC, suggesting it has the best predictive ability. In addition, a combinatorial marker panel could distinguish GDM patient from HC (AUC = 0.890). These findings suggest that the combinatorial biomarker panels have diagnostic potential for GDM. Therefore, being based on our identified biomarkers for GDM may be a new screen method for GDM.

Overall, our study had several limitations. First, the number of samples was not of a substantial amount and could have affected the stability of statistical analysis. Therefore, the conclusions of this study need to be verified by more participants. Second, all GDM patients in this study used diet control to maintain a stable blood glucose level and did not use insulin treatment, so stratified analysis could not be performed. In addition, due to the lack of records of dietary habits and physical activities and the difficulty of follow‐up, it was not possible to analyze the impact of external factors on the gut microbiota‐derived metabolites. Furthermore, we had not detected the gut microbiota and gut microbiota‐derived metabolites in the stool of GDM patients. Therefore, the levels of these compounds in the digestive tract and the main factors production cannot be fully understood. Third, we had not conducted large‐scale clinical samples verification and further molecular mechanism research. Further research is needed to clarify the relationship and interaction between these altered gut microbiota‐derived metabolites and GDM.

Taken together, using targeted metabolomics approaches, we identified 7 gut microbiota metabolites that could be novel potential biomarkers or drug treatment target for GDM, including isobutyric acid, isovaleric acid, valeric acid, caproic acid, GUDCA, THDCA + TUDCA, and LCA‐3S. The KEGG metabolic pathway had revealed that these gut microbiota‐derived metabolites were primarily involved in lipid metabolism, amino acid metabolism, and glucose metabolism. Moreover, we also found that altered metabolites of the gut microbiota were associated with dysregulated maternal glucose and lipid metabolism. Therefore, we suggest that the future of research into biomarkers and GDM is best focused on gut microbiota‐derived metabolites, which can be a novel and reliable predictor for early diagnose of GDM. However, this will require a larger sample size population‐based cohort studies for subsequent validation.

## CONFLICT OF INTEREST

The authors declare that the research was conducted in the absence of any commercial or financial relationships that could be construed as a potential conflict of interest.

## AUTHOR CONTRIBUTIONS

Rongrong Xuan and Yuping Zhou conceived and designed this study. Haimin Chen and Yajie Gao performed the experiments and analyzed the data. Jialin Li, Shuaijun Ren, and Zhenglun Yang involved in acquisition of clinical data and blood samples for this study. Yajie Gao drafted the manuscript. All authors reviewed the manuscript and approved the final version of the manuscript.

## Supporting information

Fig S1

## Data Availability

The data that supporting the findings of this study are available from the corresponding author on reasonable request.
