# Supplementary figures and images for: Alterations of gut microbiota‐derived metabolites in gestational diabetes mellitus and clinical significance
Source: J Clin Lab Anal. 2022 Mar 13;36(4):e24333. doi: 10.1002/jcla.24333 (PMC8993618; doi:10.1002/jcla.24333)

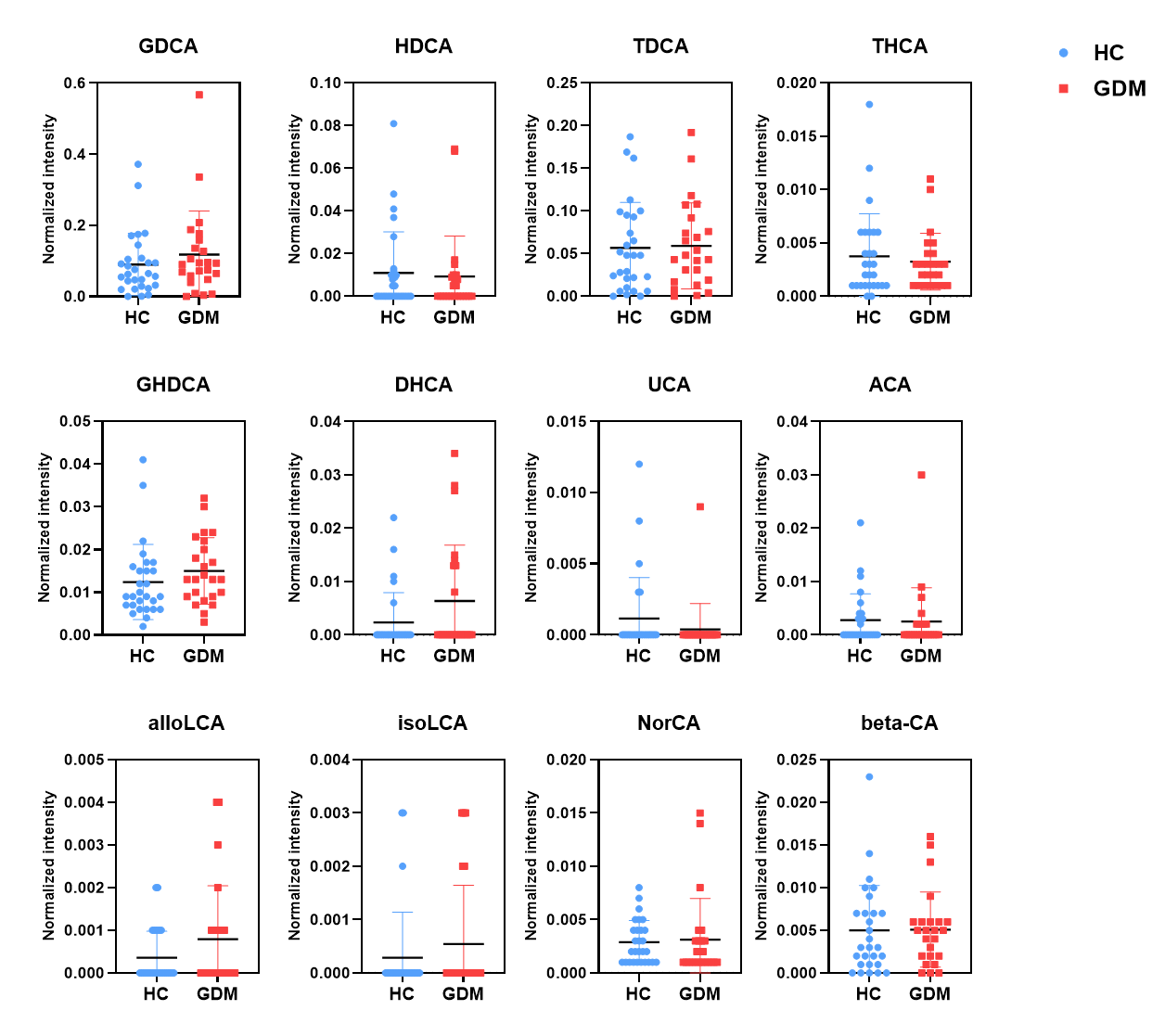

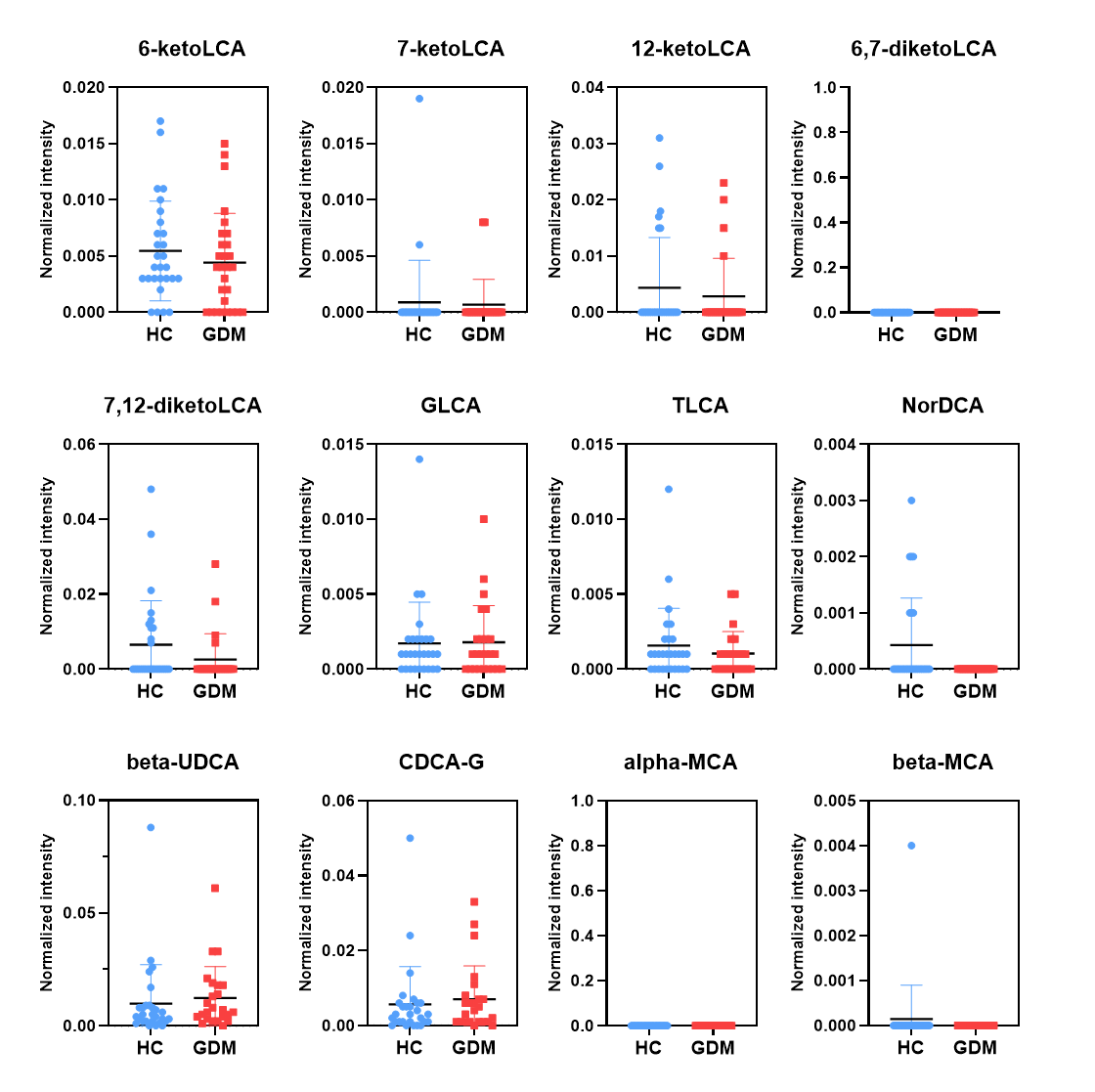


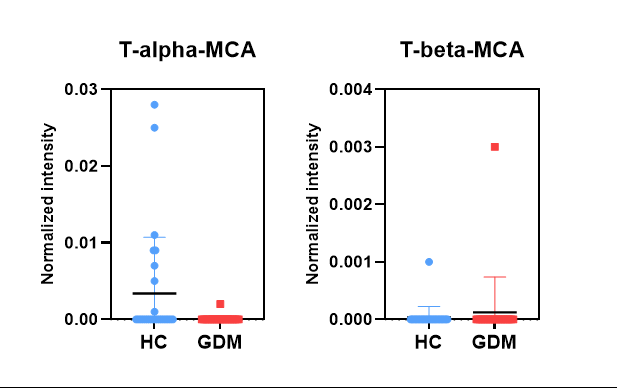


**Fig S1. Quantitative comparison of serum other BAs between GDM and HC groups**.

Supplement: Supplementary file 1 — Fig S1 [file JCLA-36-e24333-s001.docx]
